# *Serratia marcescens* outbreak in a neonatal intensive care unit associated with contaminated donor milk

**DOI:** 10.1017/ice.2022.187

**Published:** 2023-06

**Authors:** Lukas Bechmann, Ralf Böttger, Claas Baier, Aljoscha Tersteegen, Katja Bauer, Achim J. Kaasch, Gernot Geginat

**Affiliations:** 1 Department of Medical Microbiology and Infection Control, Otto-von-Guericke University Magdeburg, Germany; 2 Department of Pediatrics, Otto-von-Guericke University Magdeburg, Germany; 3 Institute for Medical Microbiology and Hospital Epidemiology, Hannover Medical School (MHH), Germany

## Abstract

**Objective:**

Investigation of the origin of a *Serratia marcescens* outbreak in a neonatal intensive care unit.

**Design:**

Retrospective case–control study.

**Setting:**

Regional level 3 perinatal center in Germany.

**Patients:**

This study included 4 *S. marcescens*–positive and 19 *S. marcescens*–negative neonates treated between February 1 and February 26, 2019, in the neonatal intensive care unit.

**Methods:**

A case–control study was performed to identify the source of the outbreak. The molecular investigation of S. marcescens isolates collected during the outbreak was performed using pulsed-field gel electrophoresis and next-generation sequencing.

**Results:**

The retrospective case–control study showed a significant correlation (P < .0001) between S. marcensens infection or colonization and consumption of donor milk that had tested negative for pathogenic bacteria from a single breast milk donor. Pulsed-field gel electrophoresis and next-generation sequencing retrospectively confirmed an S. marcescens strain isolated from the breast milk of this donor as the possible origin of the initial outbreak. The outbreak was controlled by the implementation of an infection control bundle including a multidisciplinary infection control team, temporary nutrition of infants with formula only and/or their mother’s own milk, repeated screening of all inpatients, strict coat and glove care, process observation, retraining of hand hygiene and continuous monitoring of environmental cleaning procedures.

**Conclusions:**

Low-level contaminated raw donor milk can be a source of infection and colonization of preterm infants with S. marcescens even if it tests negative for bacteria.

*Serratia marcescens* can cause nosocomial infections such as wound, respiratory, and urinary tract infections. In immunocompromised patients, it can cause bloodstream infections and meningitis with a possibly lethal outcome.^1^ *Serratia marcescens* has repeatedly caused outbreaks in neonatal intensive care units (NICUs).^2–7^ In addition to colonized neonates, reservoirs for *S. marcescens* outbreaks include the hands of staff, contaminated infant food, breast pumps and breast milk, medical devices, parenteral nutrition solutions, drugs, and care products.^8^

Donor breast milk is the preferred alternative to a mother’s own breast milk if she cannot provide enough milk to nurse her child. A valid method for inactivating relevant pathogens in donor milk is pasteurization at 62.5°C for 30 minutes using the Holder method. The use of unpasteurized donor milk is not recommended in most international guidelines for the operation of human milk banks,^9–12^ even though the pasteurization process may lead to a reduction of water-soluble vitamins and proteins involved in the immune response.^13^ In contrast to the guidelines mentioned, several German and Norwegian hospitals feed unpasteurized, low-germ donor milk due to the better immunological properties of raw donor milk and the subjective experience of better child development.^14,15^ In the former German Democratic Republic, the use of raw donor milk for the nutrition of preterm infants was widely established and is still in use in this territory.^16^

We describe an outbreak of *S. marcescens* in the perinatal center of a tertiary-care hospital, likely caused by the residual contamination of raw donor milk that had tested negative for pathogenic bacteria.

## Materials and methods

### Setting

The regional level 3 perinatal center of the University Hospital Magdeburg accommodates a 10-bed NICU and an 18-bed neonatal intermediate care unit (NIMC). Neonates in the NICU are screened weekly for methicillin-resistant *Staphylococcus aureus*, multidrug-resistant gram-negative bacteria, and bacteria with particularly high risk of nosocomial infection outbreaks (eg, *S. marcescens*) in accordance to national guidelines.^17^ Routine colonization screening of patients had not been established in the NIMC at the time of this study. Characteristics of neonates involved in the *S. marcescens* outbreak are shown in Table 1.

**Table 1.** Characteristics of Neonates Involved in the *Serratia marcescens* Outbreak

Case No.	Sex	Birthweight in Grams	Suspected Transmission Path	Infection or Colonization	Diagnosis, Days after Admission	Total Stay in Hospital, Days
1	M	1,400	DM	Colonization	30	43
2	M	550	DM	Colonization	15	98
3	F	1,220	DM, CP to case 2	BSI, meningitis, death	3	4
4	M	910	DM	Colonization	25	82
5	F	1,230	DM	BSI, full recovery	17	110
6	M	550	DM	Colonization	106	164
7	F	680	DM	Colonization	65	119
8	M	890	CP to case 2	Colonization	34	100
9	M	1,860	CP to case 4	Colonization	14	39
10	M	2,140	CP to case 7	Colonization	14	23
11	M	1,330	CP to case 7	Colonization	6	21
12	M	2,200	CP to case 7	Colonization	6	21
13	M	1,340	CP to case 7	Colonization	13	13
14	M	1,680	CP to case 4	Colonization	17	39
15	M	1,680	CP to case 4	Colonization	15	29
16	F	1,660	CP to case 7	Colonization	9	57
17	M	2,560	CP to case 4	Colonization	5	24

Note. BSI, bloodstream infection; DM, raw donor milk; CP, contact patient; M, male; F, female.

The perinatal center includes a human milk bank.^16^ Regular donors are mothers of preterm infants previously treated in the perinatal center. Screening procedures include weekly screening (nasal, throat, or rectal) of donors for multidrug-resistant bacteria. Donors collect portions of expressed milk daily, which are generally frozen at home and later transferred to the milk bank. Individual portions are thawed before use, and each portion is tested separately in the microbiology laboratory before use. All milk portions containing any potentially pathogenic bacteria (eg, *S. aureus*, Enterobacterales, nonfermenting gram-negative rods) are either pasteurized (concentration of ≤10^4^ potentially pathogenic bacteria/mL) or rejected (concentration of >10^4^ potentially pathogenic bacteria/mL; any presence of multidrug-resistant bacteria or *S. marcescens,* due to the high risk of nosocomial infection and/or limited antibiotic therapy). Detection of *S. marcescens* in single milk portions, however, did not exclude the donor completely from the donor program.

### Microbiological testing of milk samples

Milk samples (10 µL) were plated on BD Columbia agar with 5% sheep blood (Becton-Dickinson, Franklin Lakes, NJ, USA) and MacConkey-3 agar (Oxoid, Basingstoke, Hampshire, UK) and incubated at 37°C for 24–48 hours. Strain identification was performed by matrix-assisted laser desorption/ionization time-of-flight mass spectroscopy (MALDI-TOF MS).

### Colonization screening for S. marcescens

Throat and rectal swabs were plated on BD Columbia Agar with 5 % sheep blood (Becton-Dickinson) with a 10 µg colistin disk (Oxoid) placed in the first streak and plates were incubated at 37°C under a 5% CO_2_ atmosphere for 48 hours. Strain identification was performed by MALDI-TOF from all phenotypically different colonies in the colistin disk’s inhibition zone.

### Environmental investigations

Environmental surfaces were swabbed (eSwab, Copan-Diagnostics, Italy). Swabs were spun in a vortexer, and 1 mL transport medium was added to a CASO bouillon with LTHTh (Merck, Darmstadt, Germany; LTHTh containing Lecithin, Tween 80, Histidin und Thiosulfat). After 24–48 h incubation 100 µL turbid bouillon was plated onto Mac-Conkey-3-Agar (Oxoid) and ChroMedium-Coliforme-Agar (Xebios-Diagnostics, Düsseldorf, Germany) and incubated for further 24–48 hours. Bacterial identification was performed by MALDI-TOF.

### Retrospective case–control analysis

Because of the known microbiological risks of unpasteurized donor milk the microbiology records of all previously donated milk samples of all current donors were checked for *S. marcescens*. This check revealed a single “suspected” donor with previous *S. marcescens*–positive milk samples that because of the positive culture result were discarded. A retrospective case–control analysis was performed by comparing the exposure of all children treated from February 1 to February 26, 2019, in the NICU to unpasteurized milk portions of the suspected donor and other potential risk factors for *S. marcescens* infection or colonization. Cases were defined as neonates treated in the NICU who were colonized or infected with *S. marcescens* during that period. Controls were neonates treated in the NICU without evidence of *S. marcescens* colonization or infection until February 26, 2019. The exposure variables in addition to the suspected milk donor were sex, birthweight, parenteral nutrition, ventilation, the patient’s room and consumption of tea, breast milk fortifier, nystatin, and vitamin D. The χ^2^ test was used to assess significant associations between exposure and outcome variable, with a significance level of *P* < .01.

### Pulsed-field gel electrophoresis

Pulsed-field gel electrophoresis was performed according to an in-house protocol (1% agarose gel, restriction enzyme SpeI). The restriction patterns were evaluated visually based on the criteria proposed by Tenover et al.^18^

### Next-generation sequencing

DNA was isolated from overnight culture (1 colony in 5 mL LB medium) using the CTAB-lysozyme protocol by Larsen et al.^19^ Library preparation was then performed using the TruePrep DNA Library Prep Kit V2 for Illumina (1 ng) (Vazyme-Biotech, Nanjing, China) and samples were barcoded with the Nextera-XT Index Kit (Illumina, San Diego, CA, USA) according to the manufacturer’s instructions. Sequencing was performed with the MiSeq Reagent Kit version 2 (500 cycles) on the MiSeq (Illumina). Data were analyzed with Ridom SeqSphere+ software (Ridom, Münster, Germany) using a custom-made core genome with *S. marcescens* Db11 as seed genome (NZ_HG326233.1). Moreover, 6 different *S. marcescens* strains were used as query genomes: SM39, NZ_AP013063.1; WW4, NC_020211.1; CAV1492, NZ_CP011642.1; RSC-14, NZ_CP012639; SmUNAM836, NZ_CP012685.1; B3R3, NZ_CP013046.1. The samples were analyzed after de novo assembly with SKESA version 2.3.0 software.^20^ For visualization, a minimum spanning tree of the samples was created using SeqSphere+ and Mash software.^21^

## Results

### Description of the outbreak

The baseline culture positivity of *Serratia* surveillance cultures on the NICU was <1 case per year between 2014 and 2018 (mean, 0.6). In the NICU, the first 2 patients with *S. marcescens* were detected on January 19, 2019 (case 1) and February 5, 2019 (case 2) (Table 1). In both infants, rectal colonization with *S. marcescens* was detected by weekly routine screening. An epidemiological connection could not be determined. Neither infant developed an infection during their inpatient stay, and both were discharged without pathological findings. On February 25, 2019, a preterm neonate developed septicemia (case 3). The lumbar puncture showed gram-negative rods and pleocytosis in the cerebrospinal fluid. The preterm infant had been delivered 3 days earlier by caesarean section at 29/2 weeks of gestation because the mother had severe preeclampsia. The APGAR score of the infant was 7/9/9, and the infant weighed 1,220 g. Bacterial cultures of cerebrospinal fluid and blood culture yielded *S. marcescens* on the following day. Despite immediate antibiotic therapy, the infant deteriorated, suffered severe brain damage, and died the next day. On February 26, 2019, 2 further neonates (cases 4 and 5) colonized with *S. marcescens* were detected, and an outbreak situation was recognized. Of these 2 infants, case 5 subsequently developed a sepsis on day 42 of life (March 20) with *S. marcescens*–positive blood cultures but recovered completely under antibiotic therapy with meropenem. The other infant (case 4) showed no signs of infection and was discharged without pathological findings.

Exposure to potential risk factors for infection or colonization with *S. marcescens* were investigated for all children treated in the NICU from February 1 to February 26, 2019. In a retrospective case–control analysis 4 *S. marcescens*–positive neonates (cases 2–5) and 19 *S. marcescens*–negative neonates (controls) were compared (Table 2). All *S. marcescens*–positive neonates had received raw milk from a single milk donor, who was suspected as possible source because *S. marcescens* had been detected in discarded milk portions of this donor. Only 1 of 19 neonates in the *S. marcescens*–negative control group had received milk from the suspected donor (*P* < .0001). No isolate was available for case 1, so it was not included in the case–control study. As described in the materials and methods section, an aliquot of each individual milk donation was cultured for the presence of potentially pathogenic bacteria in the microbiological laboratory before feeding. The culture results of the milk donor’s 75 breast milk samples are shown in Table 3. Overall, 37 milk samples yielded only commensal bacteria from the skin without any presence of potentially pathogenic bacteria and were fed to infants unpasteurized. The lower detection level of the culture method was 100 CFU/mL. None of the 38 milk samples with growths of *Acinetobacter johnsonii*, *Escherichia coli*, or *Stenotrophomonas maltophilia* were fed to the infants without previous pasteurization. Any milk portions with growth of *S. marcescens* were discarded.

**Table 2.** Risk Factors for Colonization or Infection With *Serratia marcescens* in the Early Phase of the Outbreak (February 2019)

Group	Case Exposure		Control Exposure		*P* Value
	Yes	No	Yes	No	
Sex, male	1	3	10	9	.31
Birthweight <1.000 g	2	2	5	14	.35
Birthweight <2.000 g	4	0	11	8	.11
Parenteral nutrition	4	0	16	3	.39
Ventilation	1	3	6	13	.79
Room 3116	2	2	8	11	.77
Room 3118	2	2	4	15	.23
Raw milk of the suspected donor	4	0	1	18	<.0001
Tea	1	3	4	15	.86
Supplementation of human milk with human milk fortifier	3	1	6	13	.11
Nystatin oral	3	1	13	6	.79
Vitamin D oral	3	1	11	8	.52

**Table 3.** Microbiological Testing Results of the Suspected Milk Donor’s 75 Breast Milk Samples

Concentration of Potential Pathogenic Bacteria, CFU/mL	No. of Samples (N = 75), No. (%)	Use of Sample
None detected (<10^2^)	37 (49)	Used raw
≤10^4^ *Acinetobacter johnsonii*	7 (9)	Used after pasteurization
≤10^4^ *Escherichia coli*	1 (1)	Used after pasteurization
≤10^4^ *Serratia marcescens*	26 (35)	Discarded
>10^4^ *Serratia marcescens*	1 (1)	Discarded
≤10^4^ *Stenotrophomonas maltophilia*	3 (4)	Used after pasteurization

Note. CFU, colony-forming units.

**Fig. 1.**
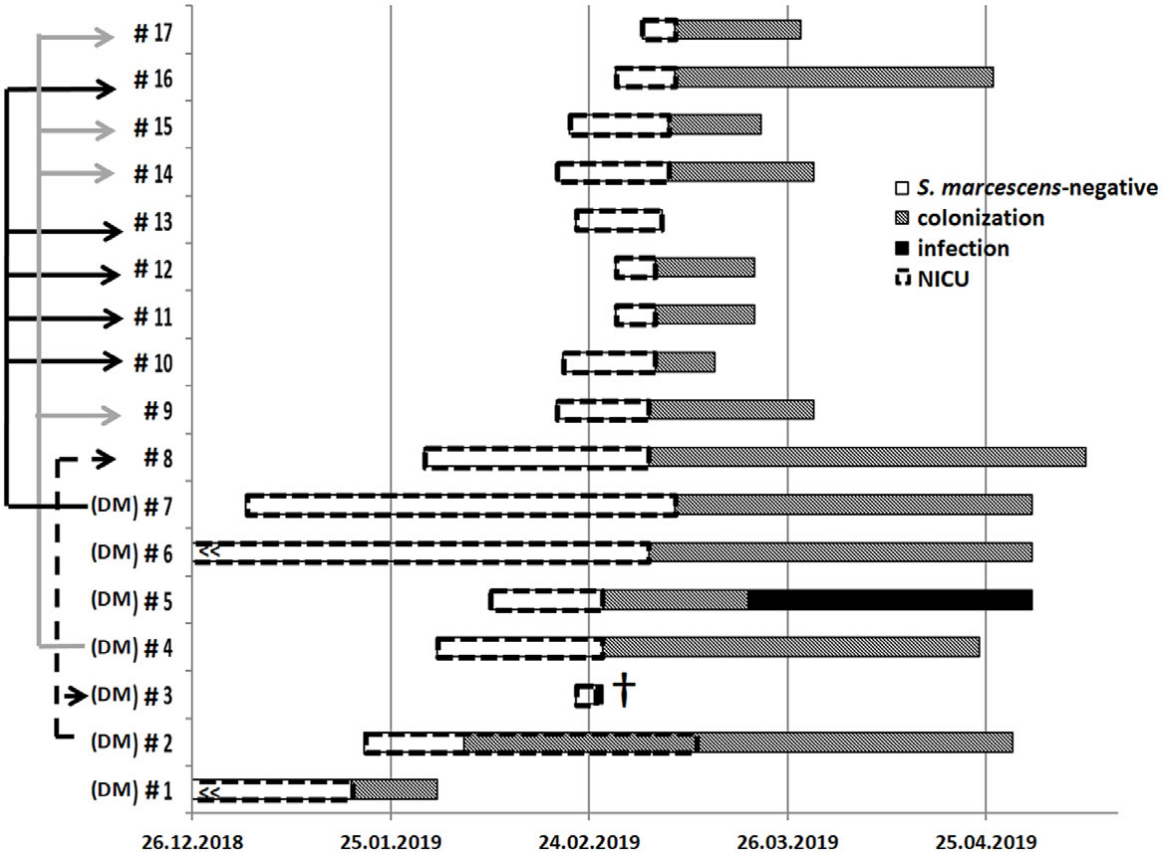
Chronology of inpatients infected or colonized with *Serratia marcescens*. Each bar represents the length of the hospital stay of an individual inpatient. Open bars indicate *S. marcescens* negative inpatients; dashed bars indicate stay at NICU; hatched bars indicate colonization with *S. marcescens*; solid bars indicate infection with *S. marcescens*, 1 inpatient died (†); DM indicates index cases 1–7 who received raw donor milk from the suspected milk donor. The arrows on the left side of the diagram indicate contact in the same room between index cases (1–7) and secondary cases (8–17) who did not receive breast milk of the suspected donor.

When the outbreak was realized in the NICU, colonization screening was extended to all NIMC patients (starting on March 5) because they had also received donor milk from the human milk bank of the hospital, and most of them were initially treated in the NICU before being transferred to the NIMC. This combined colonization screening of NICU and NIMC patients detected 12 additional infants colonized with *S. marcescens*, 1 new case in the NICU, and 11 new cases in the NIMC. None of these cases developed a *S. marcescens* infection until discharge. Of these 12 colonized infants, only 2 patients (cases 6 and 7) had received milk from the suspected donor. The other 10 cases in the NICU shared rooms with the index cases 2, 4, or 7, received care from the same staff, and were transferred to NIMC before *S. marcescens* colonization was recognized (Fig. 1).

### Infection control measures

After the outbreak was discovered, an interdisciplinary infection control team was established to coordinate further actions. After recognition of the possible association of the outbreak with raw donor milk, the further use of raw donor milk was immediately stopped, and all neonates were temporarily fed formula or their mother’s own milk. Screening for *S. marcescens* of all inpatients by rectal and throat swabs 3 times per week was initiated. As part of the environmental sampling, 85 swabs were taken in the perinatal center and in the human milk bank from frequent hand-contact surfaces in the patient rooms, medical equipment, handwashing areas, computer equipment in the patient environment, shared care utensils and medicines as well as equipment, surfaces, and supplements in the milk preparation room. *Serratia marcescens* was only detected in a single swab of a single-patient stethoscope that was used for an infant known to be colonized with *S. marcescens*. Because the use of single-patient stethoscopes was an established standard procedure on the NICU, no intervention regarding the use of stethoscopes was necessary.

To exclude any further bacterial spread from *S. marcescens*–colonized infants to *S. marcescens*–negative infants in the NICU, all colonized infants were placed in cohorts on a separate ward and were nursed by a separate team. In addition, a partial visitor stop was enforced for the whole neonatology unit, allowing visits by parents only. Empiric antibiotic treatment for suspected serious infections was switched to meropenem. The pasteurization process was revalidated. Further infection control measures included the use of disposable protective gowns and medical gloves for all medical and nursing procedures, process observation, retraining of hand hygiene and continuous monitoring of environmental cleaning procedures. After implementation of the infection control bundle, no further *S. marcescens* cases occurred in the outbreak.

### Pulsed-field gel electrophoresis and next-generation sequencing

**Fig. 2.**
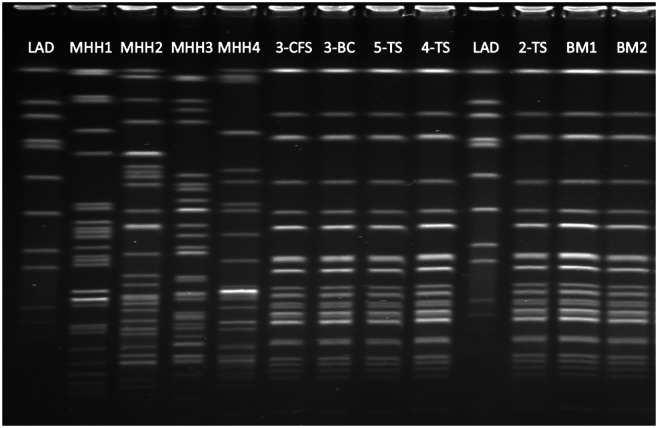
Pulsed-field gel electrophoresis of *Serratia marcescens* isolates collected during the outbreak. Note. LAD, ladder; lane MHH1/2/3/4, unrelated *S. marcescens* control isolates from the Hannover Medical School (MHH); Lane 3-CFS, case 3, isolate from cerebrospinal fluid; 3-BC, case 3, isolate from blood culture; 5-TS, case 5 isolate from throat swab; 4-TS, case 4, isolate from throat swab; 2-TS, case 2, isolate from throat Swab; BM1 and BM2, isolates from 2 breast-milk samples from the suspected donor. Case 1 was not included because no isolate was available.

Pulsed-field gel electrophoresis confirmed the identity of the *S. marcescens* isolates from the suspected donor milk and the *S. marcescens* isolates from index cases 2–5, which showed an identical banding pattern (Fig. 2).

**Fig. 3.**
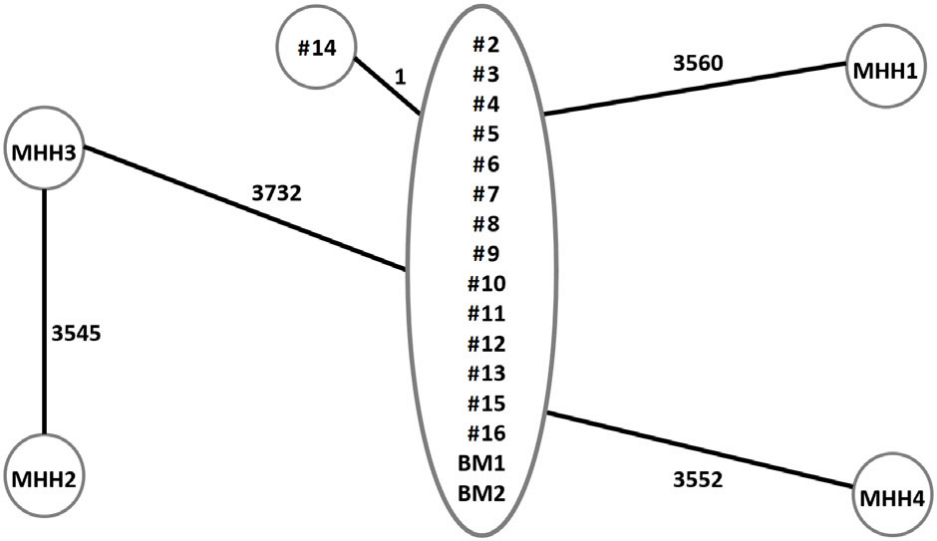
Minimum spanning tree based on core genome multilocus sequence typing (cgMLST) analysis of *Serratia marcescens* isolates from inpatients and donor breast milk. Included in the analysis were isolates from 15 inpatients (cases 2–16), 2 samples of raw donor milk contaminated with *S. marcescens* (BM1 and BM2) and *S. marcescens* isolates from the Hannover Medical School (MHH 1–4), which were included as functional controls. Isolates were put in the same circle if differences in single-nucleotide polymorphisms were not detected. The numbers on the lines between the circles indicate the genetic distance between isolates, which was calculated as the single-nucleotide polymorphism count distance between the connected samples.

For 15 of the 17 patient-derived isolates and the 2 isolates from the suspected donor milk, a whole-genome analyses were performed. Also included were 4 unrelated samples from Hannover Medical School (MHH 1–4) as controls. Only the sequence of the *S. marcescens* isolate of case 14 showed a single-nucleotide polymorphism (SNP) difference. The sequences of all other isolates including the breast milk samples showed no SNP difference, indicating a common origin of theses isolates. In contrast, the unrelated control isolates (MHH 1–4) were genetically distant from the isolates of the outbreak cluster (Fig. 3).

## Discussion

Despite several outbreaks reported in NICUs, few reports have indicated an association with the feeding of breast milk. Rettedal et al^22^ reported a clonal outbreak with a CTX-M-15 producing *Klebsiella pneumoniae* in a NICU in Norway, where 58 children were affected and 1 child developed an infection. The *K. pneumoniae* strain was probably introduced into the NICU by the breast milk of 1 mother.^22^ One probable index case was identified as the origin of the entire outbreak. All subsequent colonizations and infections probably occurred because of deficiencies in general contact precautions. In contrast to contaminated breast milk from the mother, which may result in colonization or infection of a single infant or of siblings, the use of donated milk can affect multiple infants at once. According to the report by Donowitz et al,^23^ 5 neonates contracted bloodstream infections with *K. pneumonia* after receiving contaminated raw milk from a single donor. Kato et al^24^ described an outbreak of methicillin-resistant *S. aureus* (MRSA) in a NICU in Japan. Of the 6 neonates on the ward, 5 were colonized with MRSA; 4 of these 5 cases were due to contaminated breast milk.

In other instances, outbreaks were caused by the contamination of breast milk collected from different donors by cross contamination in the human milk bank or by shared breast pumps. Gransden et al^25^ reported an outbreak of *S. marcescens* transmitted by contaminated breast pumps, which stopped after changing the disinfection procedure. Fleisch et al^26^ reported 3 consecutive outbreaks of *S. marcescens* in a NICU. The authors identified contaminated milk as the source during the third outbreak and speculated that contaminated milk played a role even in the first and second outbreaks. The colonization of neonates stopped after the reorganization of procedures in the milk kitchen.

The in-depth investigation of the outbreak described here showed that *S. marcescens* was spread among inpatients by different means. In the early phase in February 2019, the colonization or infection of infants was likely associated with the use of unpasteurized contaminated donor milk. Although any milk portions that yielded *S. marcescens* in cultures were discarded, the results of the case–control study strongly suggests that culture-negative milk portions of the suspected single donor contained viable *S. marcescens* that resulted in colonization or infection of 7 preterm infants. The cultures of these milk portions were probably negative because the bacterial concentration was below the culture detection limit of 100 CFU/mL. In response to the outbreak, the human milk inoculum for cultures in our institution was increased from 10 to 100 µL to improve the detection limit from 100 to 10 CFU/mL. Improved diagnostics, however, cannot completely exclude the risk of infection; therefore, hospitals that feed raw donor milk should always be aware that unpasteurized donor milk with low, even nondetectable colony counts could infect premature neonates. To minimize microbiological risks, most published milk-bank guidelines recommend pasteurization of donor milk.^9–12^

The later phase of the outbreak in the NIMC was suspected to have been caused by insufficient contact precautions, which may also have caused the infection of the fatal case (case 3) who temporarily stayed in a patient room with a colonized neonate. In retrospect, however, it seems equally possible that the later cases in the NIMC were caused by secondarily contaminated milk. This hypothesis is supported by observations during a second cluster of *S. marcescens* colonizations among preterm infants during August and September 2019. This second cluster was caused by a different *S. marcescens* strain, and contaminated raw donor milk did not play a direct role in the transmission of bacteria. At the time this cluster occurred, unpasteurized milk was only used if it was provided by the biological mother. Because of this situation, we realized that the risk of storing unpasteurized, potentially contaminated milk portions provided by mothers for their own babies in the same refrigerator as pasteurized milk portions for other babies. Together with insufficient contact precautions when handling the milk portions, this procedure could easily spread contaminants from the outside surface of unpasteurized milk portions. To avoid cross contamination during storage of prepared milk portions, 2 separate refrigerators were provided for each NICU and NIMC. One refrigerator was used for tested donor milk and milk for tested patients, and another was used for untested mothers’ own milk or milk for patients colonized with highly resistant bacteria or *S. marcescens*. During the second cluster, the Holder pasteurizer was upgraded to a model with complete internal documentation and traceability of process parameters, and a strict quality management system for the processing of breast milk with complete electronic documentation for easy traceability was implemented. Since implementation of these additional measures in September 2019, no further cluster of *S. marcescens* colonizations or infections has occurred.

In summary, our data suggest that raw donor milk contaminated with <100 CFU/mL of *S. marcescens* can result in the colonization and severe infection of preterm infants. Thus, more sensitive culture methods must be used to test donor milk that is to be fed without pasteurization. Further clinical studies are required to assess the inherent microbiological benefits of unpasteurized raw donor milk and the microbiological risks.^27^
